# RIG-I Has a Role in Immunity Against *Haemonchus contortus*, a Gastrointestinal Parasite in *Ovis aries*: A Novel Report

**DOI:** 10.3389/fimmu.2020.534705

**Published:** 2021-01-08

**Authors:** Samiddha Banerjee, Aruna Pal, Abantika Pal, Subhas Chandra Mandal, Paresh Nath Chatterjee, Jayanta Kumar Chatterjee

**Affiliations:** ^1^ Department of Animal Science, Visva Bharati University, Bolpur, India; ^2^ Department of LFC, West Bengal University of Animal and Fishery Sciences, Kolkata, India; ^3^ Department of Computer Science, Indian Institute of Technology, Kharagpur, India

**Keywords:** RIG-I, innate immunity, parasite, *Haemonchus contortus*, sheep

## Abstract

Retinoic acid inducible gene I (RIG-I) is associated to the DExD/H box RNA helicases. It is a pattern recognition receptor (PRR), playing a crucial role in the system and is a germ line encoded host sensor to perceive pathogen-associated molecular patterns (PAMPs). So far, reports are available for the role of RIG-I in antiviral immunity. This is the first report in which we have documented the role of RIG-I in parasitic immunity. *Haemonchus contortus* is a deadly parasite affecting the sheep industry, which has a tremendous economic importance, and the parasite is reported to be prevalent in the hot and humid agroclimatic region. We characterize the RIG-I gene in sheep (*Ovis aries*) and identify the important domains or binding sites with *Haemonchus contortus* through in silico studies. Differential mRNA expression analysis reveals upregulation of the RIG-I gene in the abomasum of infected sheep compared with that of healthy sheep, further confirming the findings. Thus, it is evident that, in infected sheep, expression of RIG-I is triggered for binding to more pathogens (*Haemonchus contortus*). Genetically similar studies with humans and other livestock species were conducted to reveal that sheep may be efficiently using a model organism for studying the role of RIG-I in antiparasitic immunity in humans.

## Introduction

The innate immune system, acting as the primary step in defense against infectious agents, recognizes the pattern recognition receptors (PRRs). They play a crucial role in the system and are germ line decoding host sensors to decipher pathogen-associated molecular patterns (PAMPs) and are expressed by innate immune cells, such as dendritic cells, macrophages, monocytes, neutrophils, and other cells. Retinoic acid inducible gene-I (RIG-I)-like receptors that are in the PRR superfamily detect viral nucleic acid in the cytosol (1). RIG-I is associated with the DExD/H box RNA helicases and is one of the members of RIG-I–like helicases; the other two are MDA5 and LGP2. RIG-I is intimately associated with the Dicer family of helicases of the RNAi pathway.

RIG-I preferably recognizes short RNA sequences marked with the 5’-triphosphate group (5’ppp) and the blunt end of short double- (ds RNA) or single-stranded RNA (ss RNA) or ss RNA hairpins (2). It binds mostly with negative strand viruses, e.g., influenza A viruses and in some positive and ds RNA viruses also (3). RIG-I is crucial for signaling influenza A, influenza B, human respiratory syncytial virus, paramyxovirus, Japanese encephalitis virus, and West Nile virus.

The molecular kinetics of RIG-I contain two N-terminal CARD domains and one RNA helicase domain, which helps in relaying the signal to the downstream signal adaptor mitochondrial antiviral signaling protein (MAVS), which tends to drive type I IFN responses. It also induces caspase-8–dependent apoptosis, preferably in tumor cells (4). Tumor cells are highly vulnerable to RIG-I–induced apoptosis although normal healthy cells are resistant to RIG-I–induced apoptosis. The RIG-I gene participates in TLR-stimulated phagocytosis (5). When RIG-I is stimulated with TLR4, it induces its expression in macrophages, and gradual depletion of RIG-I causes inhibition of TLR4-induced bacterial phagocytosis. Investigations report that RIG-I acts as a potential therapeutic target for cancer, precisely melanoma. In the context of viral infections, RIG-I induces MAVS-dependent inflammasome activation (6). Studies show that RIG-I plays a vital role in immune responses in association with different infectious and noninfectious diseases, such as astherosclerosis or skin psoriasis in which the levels of interferon gamma and RIG-I are significantly raised in the epidermis of the skin (7). RIG-I expression can be highly observed in differentiated skin and colon mucosal tissue.

Recent preliminary studies indicate the possible role of immune response genes on parasitic infections (8–10). Certain immune response genes were detected through transcriptomics analysis in the case of infection with the *Contracaecum osculatum* third-stage larvae in fish (8). Similar reports are available for the influence of the MDA5 pathway on malaria infection (9) and IRF3 on *Toxoplasma gondi* infection (10).

Indigenous sheep (*Ovis aries*) form the backbone of socioeconomically backward farmers and landless laborers and have become their way of life. Nematode infection—infection with *Haemonchus contortus*—poses a major threat to the sheep industry in both commercial farms and farmers’ herds. Earlier, we studied the genetic resistance of indigenous sheep with respect to mitochondrial genes (11). So far, information is available for the role of RIG-I on viral infection as well as a few reports on antibacterial immunity. To date, reports are not available for the antiparasitic effect of RIG-I.

Hence, the present study reflects on the information regarding the structure, function, and expression profile of the RIG-I gene in indigenous sheep through molecular methods and the role of the RIG-I gene in parasitic infection in healthy and diseased sheep for the first time.

## Materials Required and Methods

### Animals, Collection of Samples, and mRNA Isolation

#### Animals, Fecal Sample Collection, and Determination of Fecal Egg Count

We randomly collected 60 Garole sheep (*Ovis aries*) samples from the Instructional Livestock Farm Complex Farm, West Bengal University of Animal and Fishery Sciences, Mohanpur campus. The samples were collected prior to a routine deworming procedure and were presumed to have preexisting gastrointestinal parasites because they were not dewormed in the preceding 3 months during the monsoon season for the purpose of the present study. The animals were presumed to have been exposed to natural infection during grazing.

Fecal samples from the sheep were examined thoroughly by the salt floatation method (12), and the fecal egg counts (FECs) were screened for each sample. One gram of fecal samples were triturated mortar and pestle, and then 15 ml of a saturated NaCL solution was gently mixed with each fecal sample. When the mixture settled, and the upper phase of solution was collected with the help of a dropper and slowly filled a McMaster slide. The solution moved through the capillary action into the chambers and was observed under a microscope. FEC is estimated as follows: FEC = X+Y/2 x 100, where X and Y = no. of eggs in both chambers of the McMaster slide.

Based on statistical analysis, samples were collected from two groups, designated as healthy (x-bar + SD) and diseased (x-bar – SD), where x-bar stands for mean and SD means standard deviation. Sheep were regularly sold and slaughtered for the purpose of mutton production. Altogether, 12 tissue samples were screened, 6 each with low FEC (grouped as healthy) and six infected samples for further case study. Confirmation of the infected animals was also done through direct visualization of adult parasites in the abomasum. Tissue samples were collected from the abomasum, rumen, small intestine, cecum, liver, and lymph nodes.

#### mRNA Isolation

Tissues from different organs from healthy and diseased sheep were collected and subjected to total RNA isolation by the TRIzol method. These are primarily the organs of the digestive system—abomasum, rumen, small intestine, cecum, and liver—to assess gut-associated lymphoid tissue (GALT). Other tissues studied were lymph nodes, testis, urinary bladder, kidney, inguinal lymph node, and heart. Abomasum tissue was collected individually from healthy and diseased sheep (*Haemonchus contortus* affected). mRNA isolation was carried out aseptically by the TRIzol method. Each healthy and diseased abomasum tissue collected was marked and separated into the tip and the middle of the abomasum. mRNA was withdrawn from abomasum tissue by the TRIzol method and subjected to cDNA preparation (13–15). Each mixture of 2 g of tissue and 5 ml TRIzol was triturated, followed by chloroform treatment. Centrifugation resulted in three-phase differentiation from which the aqueous phase containing the mRNA was separated. It was treated by isopropanol to extract the mRNA in the form of a pellet, discarding the supernatant as a result of centrifugation. Finally, the pellet underwent an ethanol wash and was air-dried, followed by dissolving the mRNA in nuclease-free water. RNA concentration and quality was estimated by nanodrop as per standard procedure.

#### Materials Required

10X buffer, dNTP, and Taq DNA polymerase were purchased from Invitrogen; SYBR Green qPCR Master Mix (2X) was purchased from Thermo Fisher Scientific Inc. (PA, USA). Primers were purchased from Xcelris Labs Limited. The reagents used were of analytical grade.

### Amalgamation, Configuration of cDNA, and PCR Amplification of RIG-I Gene

First, 20 μL reaction mixture volume consisted of 5 μg of complete RNA, 40 U of ribonuclease inhibitor, 0.5 μg of oligo dT primer (16–18 mer), 1000 M of dNTP, 5 U of MuMLV reverse transcriptase in reverse transcriptase buffer, and 10 mM of DTT. The reaction mixture was carefully blended and incubated properly at 37°C for 1 h. The reaction was ceased and heated at 70°C for 10 min and then immediately chilled on ice. The quality of the cDNA was checked by polymerase chain reaction. To obtain a full-length open reading frame (ORF) of the gene sequence, specific primers pairs were designated for the amplification of RIG-I and confirmed based on the mRNA sequences of *Bos indicus* by DNASTAR software as in **Table 1**. The amplified products were the overlapping sequences joined to extract the full sequence.

**Table 1:** Primers used for amplification of RIG-I gene of Garole sheep as overlapping sequences.

Gene	Primer	Length	Tm
**RIG-I.1**	**FP :** CAGGCATGACGGCAGAGCAGCG **RP :** TTGATAATGAGGGCATCATTGTATTTCCGA	22 31	62 59
**RIG-I.2**	**FP :** GTGTTTCAGATGCCAGACAAAGAGGAAGA **RP:** TCTTCCTCTGCCTCTGGTCTGGATCAT	29 27	60 61
**RIG-I.3**	**FP:** GTCGCCGATGAAGGCATTGACATTGC **RP:** CCTGAGCCCAAGGGGACATTTCTGC	26 25	61 63

### Sequence Analysis

The nucleotide sequence so extracted was scrutinized for protein translation, contigs comparisons, and sequence alignments by DNASTAR Version 4.0, Inc., USA. Novel sequences were submitted to the NCBI Genbank and accession numbers were obtained and are available in the public domain (Accession no. KX687005).

### Study of Predicted Ovine RIG-I Peptide Using Bioinformatics Tools

The edited peptide sequence of the RIG-I gene of Garole sheep was obtained by Lasergene Software, DNASTAR, and then alignment of the RIG-I peptide of other rodent species and *Homo sapiens* using MAFFT (16) was performed.

Prediction of the signal peptide of the RIG-I gene was derived by using the Signal P 3.0 Sewer-prediction results software, Technical University of Denmark. Calculation of leucine percentage was done manually from the predicted peptide sequence. Disulfide bonds were derived using proper and suitable software (http://bioinformatics.bc.edu/clotelab/DiANNA/) and homology searching with other species (17).

Detection of leucine-rich nuclear export signals (NES) was done with the NetNES 1.1 Server, Technical University of Denmark. O-linked glycosylation site analysis was carried out using the NetOGlyc 3.1 server (http://www.expassy.org/), whereas N-linked glycosylation detection was done by NetNGlyc 1.0 software (http://www.expassy.org/). Protein sequence–level analysis was carried out by Blast (http://www.expasy.org./tools/blast/) for determination of leucine-rich repeats (LRR), leucine zipper, N-linked glycosylation sites, detection of leucine-rich NES, and detection of the position of the GPI anchor.

α-helix and β-sheet regions were predicted using NetSurfP-Protein Surface Accessibility and Secondary Structure Predictions, Technical University of Denmark (18). Detection of leucine zipper was obtained through Expasy software, Technical University of Denmark. Domain linker prediction was carried out according to the software developed (19). LPS binding and signaling sites are essential factors for innate immune function such as pathogen recognition and binding; LPS-binding and LPS-signaling sites (20) were predicted based on homology studies with other species CD14 polypeptide.

### Model Quality Assessment and 3-D Structure Prediction

The templates that contain the highest sequence identity with our target template were identified by using PSI-BLAST (http://blast.ncbi.nlm.nih.gov/Blast). The PHYRE2 server (21) was used for homology modeling, and a 3-D structure based on homologous template structures was detected. Subsequently, the mutant model was generated using the PyMoL tool. The Swiss PDB Viewer was employed for controlling energy minimization. The 3-D structures were analyzed by PyMOL (http://www.pymol.org/), which is an open-source molecular visualization tool. The structural evaluation along with stereochemical quality assessment of the predicted model were obtained by using the Structural Analysis and Verification Server (SAVES), which is an integrated server (http://nihserver.mbi.ucla.edu/SAVES/). The Protein Structure Analysis (ProSA) web server (https://prosa.services.came.sbg.ac.at/prosa) was used for clarifying and validation of the protein structure. ProSA was used for overall perspective and checking the model structural quality for potential errors; the program shows a plot of its residue energies and Z-scores, which determine the overall quality of the model (22). TM align software was used for 3-D structure alignment of IR protein for different species and RMSD prediction to generate the structural differentiation (23). The solvent accessibility surface area of the IR genes was generated by using NetSurfP server (http://www.cbs.dtu.dk/services/NetSurfP/) (20). It calculates relative surface accessibility, Z-fit score, probability for Alpha-Helix, probability for beta-strand and coil score, etc.

### Protein–Protein Interaction Network Depiction

In order to analyze the network of the RIG-I peptide, we performed analysis by submitting FASTA sequences to STRING 9.1 (24). In STRING, the functional interlinkage was analyzed by using a confidence score. Interactions with a score <0.3 are considered low, scores ranging from 0.3 to 0.7 are classified as medium, and scores >0.7 yield high confidence. The functional partners are illustrated.

### Molecular Docking

Molecular docking is a bioinformatics tool used for in silico analysis for the prediction of binding modes of a ligand with a protein 3-D structure. Patch dock is an algorithm for molecular docking based on the shape complementarities principle (25).

### Differential mRNA Expression Profiling of Ovine RIG-I With Real-Time PCR (qRT-PCR)

#### Differential mRNA Expression Profile Was Conducted in Two Phases

Differential mRNA expression profiling of the RIG-I gene was conducted for different organs of sheep in the first phase. These are the abomasum, duodenum, rumen, cecum, and liver among the digestive system. Other organs are the heart, kidney, urinary bladder, lymph node, testis, and inguinal lymph node. RIG-I expression was observed to be important for GALT. RIG-I was expressed mostly in the abomasum of sheep.

Considering the above findings, and because *Haemonchus* sp. mostly infects the abomasum, in second phase, we studied differential mRNA expression profiling of the abomasum in healthy and *Haemonchus* infected sheep.

All qRT-PCR reactions were conducted on an ABI 7500 system. An equal amount of RNA (quantified by a Qubit fluorometer, Invitrogen), wherever required, was used for cDNA preparation (Superscript III cDNA synthesis kit; Invitrogen). Each reaction mixture consisted of 2 µl cDNA, 10 µl of single strength SYBR Green PCR Master Mix, 0.5 µl of each forward and reverse primers (10 pmol/µl), and nuclease-free water for a final volume of 7 µl. Each sample was run in duplicate. Analysis of qRT-PCR was performed by the delta-delta-Ct (ΔΔCt) method. The list of primers used for QPCR study is listed in **Table 2**:

**Table 2:** List of primers used for QPCR study.

Gene	Primer sequence
**18S rRNA F**	5′-GAGAAATTGTGCGTGACATCA-3′
**18S rRNA R**	5′-CCTGAACCTCTCATTGCCA-3′
***RIG-I F***	5′- CTTGCAAGAGGAATACCACTTAAACCCAGAGAC -3′
***RIG-I R***	5′- TTCTGCCACGTCCAGTCAATATGCCAGGTTT -3′

### Phylogenetical Analysis

Nucleotides as well as derived amino acid sequences were then aligned with that of the reported sequences of different species derived from Gene Bank (http://www.ncbi.nlm.nih.gov/blast) for the RIG-I gene. Phylogenetic analysis was conducted with MAFFT software to determine the evolutionary relationship. A neighbor-joining method was employed to reconstruct phylogeny for the putative alignment with MAFFT (16) after the multiple alignment was completed.

### Statistical Analysis

Computational descriptive statistics were done through Microsoft Excel ANOVA. In addition, it was used to test between groups and intervals between hours. Fischer’s restricted least significant differences criterion was used to keep a prior type I error rate of 0.05. All statistical analyses were conducted out using SYSTAT 13.1 software (SYSTAT Software Inc.).

## Results

### Assessment of Parasitic Infection in Sheep

Screening of the FEC of the sheep (*n*=60) led to study of the health condition of the sheep, which further led us to divide them into two categories: healthy and diseased groups. The mean FEC of the animals are given in **Table 3**.

**Table 3:** Mean FEC of the Healthy and Diseased sheep.

Health Status of the sheep	Mean Fecal Egg Count
Healthy	50 ± 5.56
Disease	550 ± 9.45

### Molecular Characterization of RIG-I Gene of Sheep

**Figure 1:**
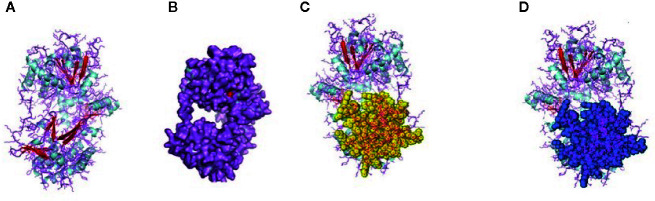
**(A)** 3-D Structure of RIG-1 gene of sheep. **(B)** 3-D Structure of RIG-1 gene (surface view) of sheep. **(C)** RIG-I_C-RD, the C-terminal domain of RIG-I of sheep. **(D)** RIG-I_C, C-terminal domain of RIG-I protein of sheep.

The RIG-I gene of sheep was characterized, and the sequence obtained was submitted to Gene Bank (accession no. KX687005). The 3-D structure is depicted in **Figure 1A**. The surface view of the RIG-I gene is given in **Figure 1B**, which was extracted from the PyMol software. Using different bioinformatics tools, several important domains of the gene and their functions are listed in **Table 4**:

**Table 4:** List of the important domains of RIG-I of sheep assessed through *in silico* study.

RIG-I_C-RD	C- terminal domain of RIG-I	772-886	**Figure 2**
RIG-I_C	C-terminal domain of Retinoic acid-inducible gene (RIG)-I protein	769-882	**Figure 3**
CARD_RIG-I_r1	Caspase activation and recruitment domain found in RIG-I	2-92	
CARD_2	Caspase recruitment domain	1-93	
RLR_C	C-terminal domain of Retinoic acid inducible gene-I	771-882	**Figure 4**
RLR_C_like	C-terminal domain of Retinoic acid-inducible gene	772-856	**Figure 5**
CARD_RIG-I_r2	Caspase activation and recruitment domain found in RIG-I	100-160	
CARD_IPS-1_RIG-I	Caspase activation and recruitment domains (CARDs) found in IPS-1 and RIG-I-like RNA helicases	2-92	
CARD_2	Caspase recruitment domain	99-160	
LGP2_C	C-terminal domain of Laboratory of Genetics and Physiology 2 (LGP2)	771-883	**Figure 6**
MDA5_C	C-terminal domain of Melanoma differentiation-associated protein 5	772-883	**Figure 7**
CARD_IPS-1_RIG-I	Caspase activation and recruitment domains (CARDs) found in IPS-1 and RIG-I-like RNA helicases	100-183	
CARD_MDA5_r1	Caspase activation and recruitment domain found in MDA5, first repeat; Caspase activation	12-81	
MPH1	ERCC4-related helicase (Replication, recombination and repair)	678-769	**Figure 8**
CARD_RIG-I_r2	Caspase activation and recruitment domain found in RIG-I, second repeat	16-93	
CARD_MDA5_r2	Caspase activation and recruitment domain found in MDA5, second repeat	41-87	
CARD	Caspase activation and recruitment domain: a protein–protein interaction domain	6-87	**Figure 9**
PRK13766	Hef nuclease; Provisional	678-771	**Figure 10**

First, the RIG-I_C-RD domain (772-886), the C-terminal domain of RIG-I, is shown as the yellow portion in **Figure 1C**. RIG-I_C(769-882), the C-terminal domain of RIG-I protein is shown as a blue portion in **Figure 1D**.

**Figure 2:**
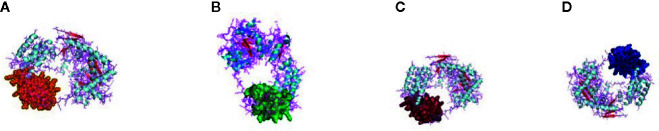
**(A)** RLR_C-terminal domain of the gene of sheep. **(B)** RLR_C_like domain of RIG gene of sheep. **(C)** LGP2_C, C-terminal domain of Laboratory of Genetics and Physiology 2 (LGP2) of sheep. **(D)** MDA5_C, C-terminal domain of melanoma differentiation-associated protein 5 of sheep.

**Figure 3:**
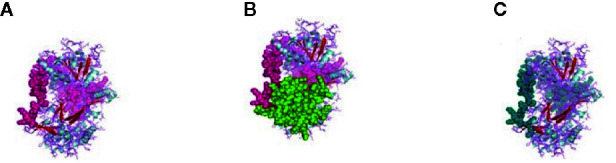
**(A)** MPH1 ERCC4-related helicase (replication, recombination and repair) of sheep. **(B)** CARD: a protein–protein interaction domain of sheep. **(C)** PRK13766, Hef nuclease; Provisional.

The CARD_RIG-I_r1(2-92) is a caspase activation and recruitment domain found in RIG-I. CARD_2 (1-93), which is also a caspase recruitment domain is present somewhat in the similar portion. RLR_C (771-882) is a C-terminal domain of RIG-I depicted in **Figure 2A** as an orange color portion. The RLR_C_like domain (772-856) C-terminal domain of RIG-I in **Figure 2B** is depicted in the green color part. CARD_RIG-I_r2 (100-160) is the caspase activation and recruitment domain found in RIG-I. Here again, the 2-92 portion is the CARD_IPS-1_RIG-I domain, i.e., CARDs found in IPS-1 and RIG-I-like RNA helicases. CARD_2 (99-160) is the caspase recruitment domain of the gene. LGP2_C (771-883), which is in the brown color in **Figure 2C** is the C-terminal domain of Laboratory of Genetics and Physiology 2 (LGP2). **Figure 2D** highlighted with the deep blue color is the MDA5_C (772-883), that is the C-terminal domain of melanoma differentiation-associated protein 5. CARD_IPS-1_RIG-I (100-183) is the CARDs found in IPS-1 and RIG-I-like RNA helicases. CARD_MDA5_r1 (12-81) represents the CARD found in MDA5, first repeat, caspase activation. MPH1(678-769) showing in the pinkish color in **Figure 3A** is the ERCC4-related helicase, the functional domain that helps in replication, recombination, and repair.

CARD_RIG-I_r2(16-93) is the second repeat of the domain CARD found in RIG-I. Similarly, CARD_MDA5_r2(16-93) is the second repeat of the CARD found in MDA5. CARD domain (6-87) is depicted in **Figure 3B** in the green and pink portion and is the CARD that functions as a protein–protein interaction domain. Last, the PRK13766 (678-771) is the Hef nuclease provisional domain in a bluish color in **Figure 3C**.

### Depiction of Protein–Protein Interaction Network and Estimation of Biological Function

**Figure 4:**
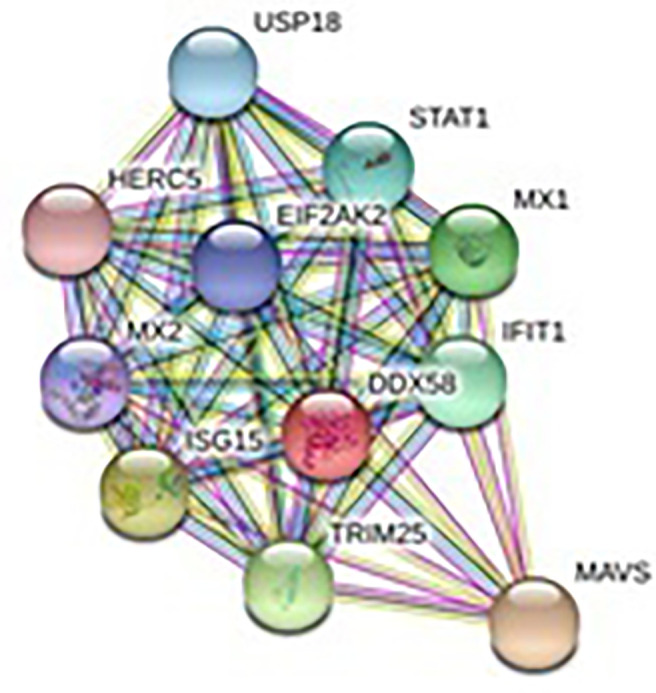
Protein–protein interaction network of RIG-I gene with associated protein by STRING analysis.

The relationship of RIG-I(DDX58 or DExD/H-box helicase 58) to other proteins has been obtained by String analysis (**Figure 4**). The related proteins revealed are MAVS (520 aa), ISG15 (a ubiquitin-like protein that has an essential role in the innate immune response to viral infection either *via* its conjugation to a target protein, ISGylation, or *via* its action as a free or unconjugated protein). ISGylation has a flow of enzymatic reactions involving E1, E2, and E3 enzymes, which catalyze the conjugation of ISG15 to a lysine residue in the target protein that exhibits antiviral activity toward both DNA and RNA viruses. The secreted form of ISG15 can persuade natural killer cell proliferation and induce lymphokine-activated-killer (LAK) activity. Tripartite motif containing 25 (TRIM25), mRNA (631 aa), MX1 (interferon-induced GTP-binding protein Mx1), interferon-induced dynamin-like GTPase show antiviral activity against rabies virus (RABV), vesicular stomatitis virus (VSV), and murine pneumonia virus (MPV). Isoform 1 but not isoform 2 depict antiviral activity against VSV and belongs to the TRAFAC class dynamin-like GTP ase superfamily. Dynamin/Fzo/YdjA family (651 aa), IFIT1 (interferon-induced protein with tetratricopeptide repeats 1 (474 aa), STAT1 (signal transducer and activator of transcription 1, 91kDa, mRNA (1162 aa), USP18 (ubiquitin specific peptidase 18) belongs to the peptidase C19 family, EIF2AK2 (Eukaryotic translation initiation factor 2- alpha kinase 2), MX2 (interferon-induced GTP-binding protein Mx2, interferon-induced dynamin like GTPase with antiviral activity against VSV, HERC5 (hect domain and RLD 5).

**Figure 5:**
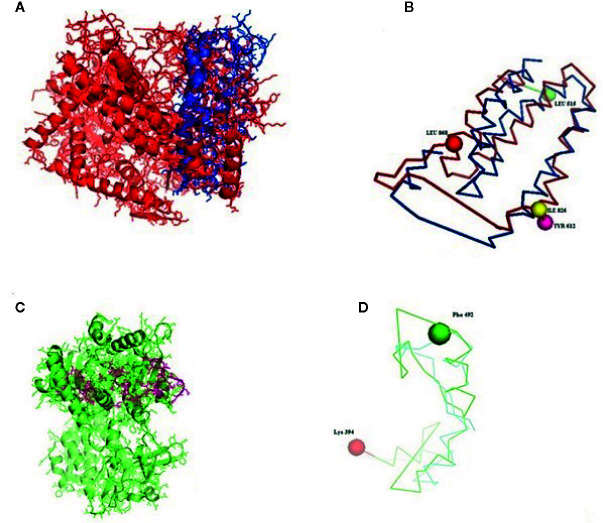
**(A–D)** Molecular docking of parasitic protein nd_4_ with ovine RIG-I, alignment (left) and binding site (right).

**Figure 6:**
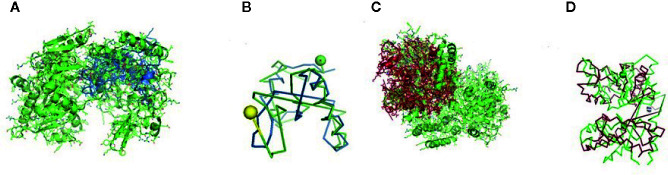
**(A, B)** Molecular docking of parasitic protein 15 kDa secretory protein with ovine RIG-I, alignment (left) and binding site (right). **(C,D)** Molecular docking of parasitic protein Pgp1 with ovine RIG-I, alignment (left) and binding site (right).

**Figure 7:**
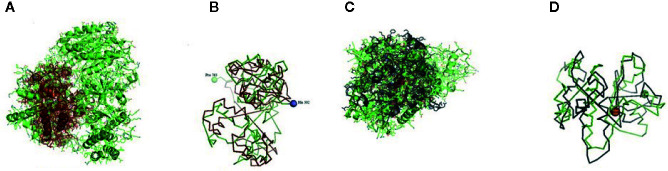
**(A, B)** Molecular docking of parasitic protein Alpha tubulin with ovine RIG-I, alignment (left) and binding site (right). **(C, D)** Molecular docking of parasitic protein Glc 5 with ovine RIG-I.

**Figure 8:**
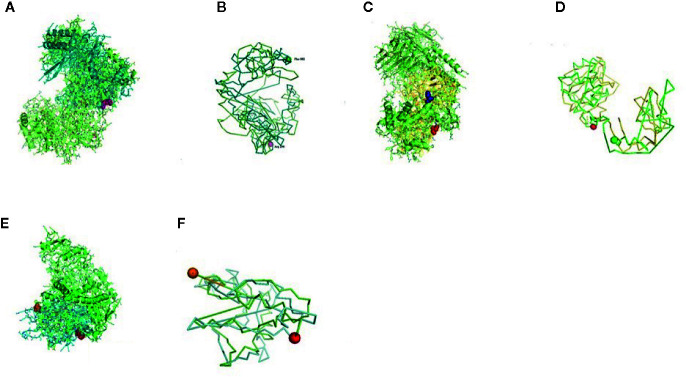
**(A)** Molecular docking of parasitic protein Beta tubulin with ovine RIG-I, alignment (left) and binding site (right). **(B)** Molecular docking of parasitic protein Phosphoenol pyruvate carboxy kinase with ovine RIG-I, alignment (left) and binding site (right). **(C)**: Molecular docking of parasitic protein Cystein proteinase with ovine RIG-I, alignment (left) and binding site (right). **(D, E)** Molecular docking of parasitic protein Galaectin with ovine RIG-I, alignment (left) and binding site (right). **(F)** Molecular docking of parasitic protein Lectin with ovine RIG-I, alignment (left) and binding site (right).

### In Silico Analysis for the Detection of the Binding Site of RIG-I With Parasite (Haemonchus contortus)—Molecular Docking

Some of the important structural proteins of *Haemonchus contortus* are identified and sequences retrieved from gene bank (NCBI). The current section (**Table 5**) discusses the findings obtained from molecular docking of RIG-I with identified domain of H. contortus with the prediction of possible binding sites.

**Table 5:** Molecular docking analysis of RIG-I with the protein of *Haemonchus contortus*.

Serial No.	Parasitic protein	Binding site	Alignment	Function of the protein
1	nd_4_	515 leucine (green sphere) to 612 tyrosine (hot pink) (**Figures 5A, B**) 826 isoleucin (yellow) to 868 leucine (red) (**Figures 5C, D**)	RIG-I (green)	Core subunit of the mitochondrial membrane respiratory chain NADH dehydrogenase (Complex I). Ubiquinone activity.
2	Pgp1	Lys 394 (red sphere) to Phe 492 (green sphere) (**Figures 6C, D**)	RIG-I (green) Pgp (Orange)	Function as efflux pumps, removing lipophilic xenobiotic compounds from cells. Play a role in the resistance of parasitic nematodes to anthelmintic drugs, such as benzimidazoles and macrocyclic lactones
3	Secretory	Val 520 (green sphere) to Asn 665 (yellow sphere) (**Figures 6A, B**)	RIG 1 (green)	Group of protein dominates the immune system of the host
4	Glc 5 (Glutamate gated chloride channel)	Gly 307 (warm pink) to Leu 868 (green sphere) (**Figure 7C**)	RIG-I(green) Glc 5 (Purple) (**Figure 7D**)	Extra cellular ligand gated ion channel activity, ion transport, transmembrane signaling activity
5	Alpha tubulin	His 302 (green sphere) to Proline 763 (Purple blue sphere) (**Figure 7B**)	RIG-I (green) Alpha tubulin (fire brick red) (**Figure 7A**)	Major constituent of microtubules. It binds two moles of GTP, one at an exchangeable site on the beta chain and one at a nonexchangeable site on the alpha chain.
6	Beta tubulin	His 302 (green sphere) – Pro 763 (green sphere) (**Figure 8A**)	RIG-I (green) Beta tubulin (Chocolate)	Major constituent of microtubules. It binds two moles of GTP, one at an exchangeable site on the beta chain and one at a nonexchangeable site on the alpha chain.
7	Cystein proteinase	Met 305 (red ruby sphere) to Glu 748 (green sphere) (**Figure 8C**)	RIG-I (green) Cystein proteinase (gray)	Cystein type peptidase activity
8	Phosphoenol pyruvate carboxy kinase	Arg 298 (hot pink sphere) to Phenyl alanine 882 (Green) (**Figure 8B**)	RIG-I (green) PEPK (deep)	GTP binding Metal ion binding Phosphoenol pyruvate carboxy kinase (GTP) activity In parasitic nematodes PEPCK carboxylates phosphoenolpyruvate to oxaloacetate, thus introducing the products of glycolysis to mitochondrial metabolism. Catalyzes the conversion of oxaloacetate (OAA) to phosphoenolpyruvate (PEP).
9	Galaectin	Threonine 330 (red sphere) to Leucine 717 (green sphere) (**Figure 8E**)	RIG-I (green) Galactin (yellow orange) (**Figure 8D**)	Carbohydrate binding
10	Lectin	Histidine 295 (red sphere) – Threonine 462 (orange sphere)	RIG-I (green) Lectin (cyan) (**Figure 8F**)	Proteins that recognize and bind specific carbohydrates

The table shows the binding sites of the *Haemonchus* sp. protein with the RIG-I protein analyzed by molecular docking, binding of RIG-I of sheep with ND4 domain of *Haemonchus contortus*.

The binding positions of the parasitic protein nd4 has been found in between 515 leucin (green sphere) to 612 tyrosine (hot pink) and 826 isoleucin (yellow) to 868 leucine (red). The pgp1 protein that binds from Lysine 394 (red sphere) to Phenylalanine 492 (green sphere) possesses ATPase activity. Several secretory parasitic proteins that play an important role in dominating the host immune system have also been observed at the position between Valine 520 (green sphere) and Asparagine 665 (yellow sphere). The Glc 5 parasitic protein (Glutamate-gated chloride channel) binds from Glycine 307 (warm pink) to Leucine 868 (green sphere). α- and β-tubulin, being major constituent of microtubules, bind two moles of GTP, one at a nonexchangeable site on the alpha chain and one at an exchangeable site on the beta chain. They bind from the Histidine 302 (green sphere) position to Proline 763 (Purple blue sphere) position. The parasitic protein Cystein proteinase binds from the Methionine 305 (red ruby sphere) position to Glutamate 748 (green sphere). Phosphoenol pyruvate carboxy kinase is yet another important parasitic protein that binds from the position Arginine 298 (hot pink sphere) to Phenylalanine 882 (Green). Last, Galaectin from position Threonine 330 (red sphere) to Leucine 717 (green sphere) and Lectin from position Histidine 295 (red sphere) and Threonine 462 (orange sphere) are the proteins that recognize and bind to specific carbohydrate moieties.

### Differential mRNA Expression Profile of Garole Sheep With Respect to RIG-I Gene in Healthy Sheep With Respect to Different Body Tissues

**Figure 9:**
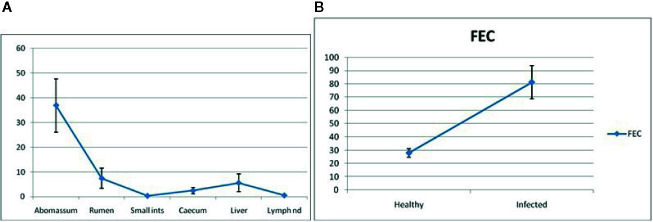
**(A)** RIG-I expression profiling for different GALT and lymph node in sheep. **(B)** RIG-I expression profiling for healthy and Haemonchus contortus–infected sheep from abomasum.

**Figure 9A** depicts RIG-I expression profiling for different GALTs and lymph nodes. It shows the expression of mRNA to be highest in the abomasum tissue almost fivefold more than all the other organs. Because we are studying RIG-I gene expression in GALT in response to *Haemonchus contortus* infection, it is observed from **Figure 9A** that expression is least in lymph nodes and small intestine. Highest is observed in abomasum followed by rumen, then liver. Cecum reveals comparatively less expression (**Figure 9A**).

### mRNA Expression Profile of Garole Sheep With Respect to RIG-I Gene in Healthy Sheep and Those Infected With Haemonchus contortus

RIG-I expression profiling for healthy and *Haemonchus contortus*–infected sheep from abomasum is clearly shown in **Figure 9B**. The assessment of differential mRNA expression profiles of RIG-I genes in Garole sheep with respect to healthy and infected sheep is represented in the figure. RIG-I is better expressed in infected sheep compared to in healthy sheep by about 2.5-fold.

### Molecular Evolution of Sheep With Other Species With Respect to RIG-I Gene

**Figure 10:**
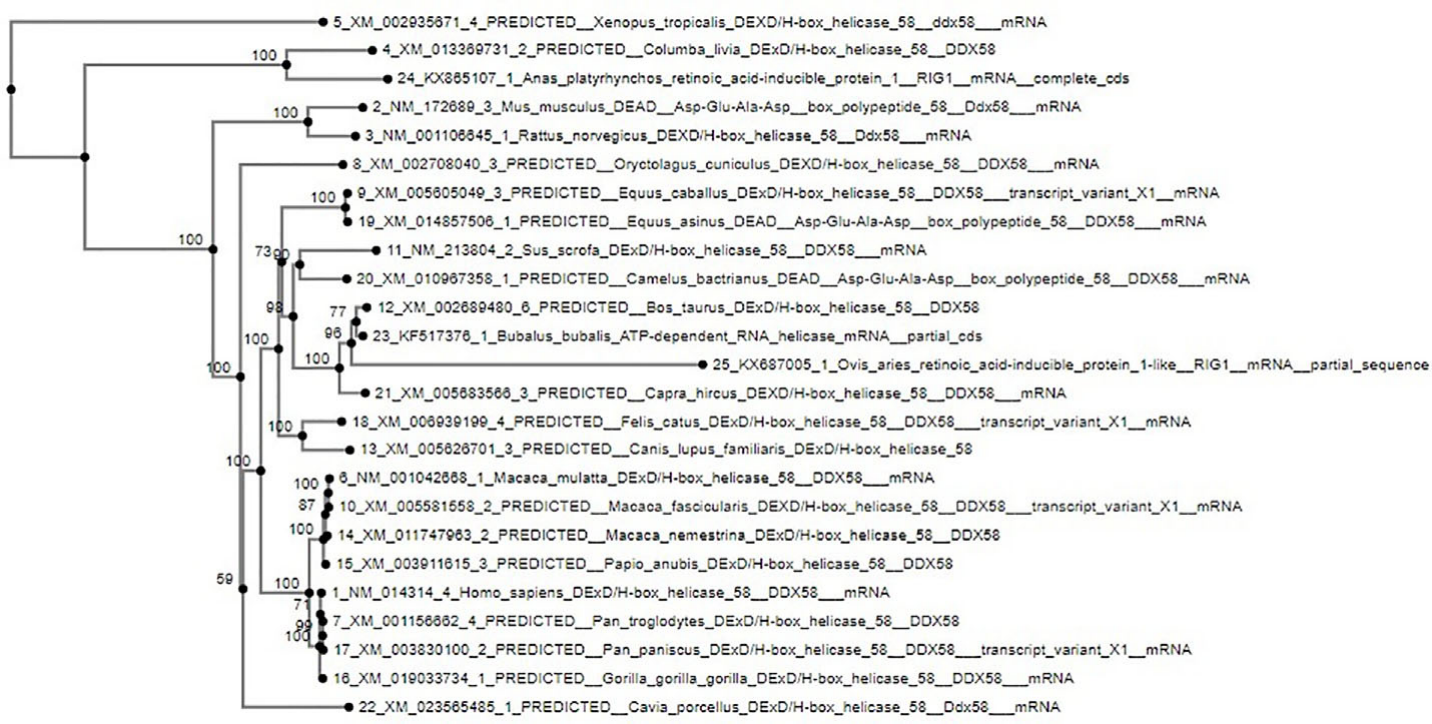
Molecular phylogenetic analysis of sheep with other species based on RIG-I gene.

The molecular phylogenetic analysis of sheep has been conducted with humans, other primates, and livestock species as depicted in **Figure 10** with respect to the gene of interest. All the ruminant species are clustered together (goat, cattle, and buffalo). Next closely related species are pig and camel. The tree depicts a closer genetic relationship of humans with sheep compared to that of lab animals, such as rats or mice.

## Discussion

RIG-I, which is considered to be a family of the RLR family, is known to recognize viral DNA in the cytoplasm. According to previous findings and information, RIG-I was considered to act as an antiviral gene, which recognizes double-stranded viral DNA and plays an important role in immune regulation. CARD-like regions are present in the gene at its N-terminal position, which acts as an interacting domain with other proteins in which CARD regions are present. It also consists of a repressor domain at the C-terminal position, which takes part in RNA binding and the central DExD helicase domain that acts as the ATP binding motif. RIG-I is reported to have a role in immune responses in association with different infectious and noninfectious diseases, such as astherosclerosis and skin psoriasis in which the levels of interferon gamma and RIG-I is significantly raised in the epidermis of the skin (7).

Previously, much less was known about the role of the RIG-I gene as an antiparasitic in sheep; therefore, in our experiment, we tried to highlight the fact that RIG-I can be induced in response to parasitic infection and can influence the immune regulation against it.

RIG-I expression is observed to be important for GALT according to some reports. It is an important component of musoca-associated lymphoid tissue, which regulates the immune system of the body and protects the gut from the invasion of infections although, being a component of the immune system, its fragility and permeability makes it vulnerable to infecting agents. Mostly, parasites invading the animal find a way through this route in the body. GALT consists of several plasma cells that can produce several antibodies, which act as a defense against GI nematodes. *Haemonchus* sp., being a fatal blood-sucking GI nematode affects the mucus membrane that affects the abomasum portion of the sheep (26).

In the present work, we have randomly categorized the sheep into two groups—healthy and diseased—based on the examination of the FEC. Several earlier studies were conducted on this particular gastrointestinal nematode, Haemonchus contorts, on sheep; however, they were restricted mostly to biochemical and hematological analysis, not molecular, based on both natural infection and artificial challenge studies with higher confidence level (27–30). Sixty sheep were taken into account from which six in number from each group with the highest and lowest fecal counts were considered for the statistical analysis. Here, we show differential mRNA expression profiling of abomasum in healthy and Haemonchus-infected sheep and observe that the differential mRNA expression level for RIG-I was much higher (2.5-fold) in Haemonchus-infected sheep in comparison with that of healthy subjects. The abomasum reveals a marked increase in lymphoid tissue compared to lymph nodes. Because lymph nodes are a secondary lymphoid organ, they play a role in the adaptive immune system. Due to better expression levels and because Haemonchus affects the abomasum severely, we consider this part to be the most affected part, which would show a proper level of gene expression.

This implies the better expression of RIG-I in diseased sheep, and thus, it is involved in immune response. This is the first report of the role of RIG-I in parasitic immunity. To establish the fact and to predict the binding site, we analyzed with molecular docking for RIG-I with different proteins (mostly surface protein) of Haemonchus contortus. Becaue this is the first report, the mechanism of action of RIG-I is not known against parasitic diseases. Earlier reports indicate that, when RIG-I is stimulated with TLR4, it induces its expression in macrophages, and gradual depletion of RIG-I causes inhibition of TLR4-induced bacterial phagocytosis (31). Likewise, genes analogous with the early inflammatory response and even those encoding toll-like receptors, such as TLR 2, 4, and 9 or in close relationship with free radical production (DUOX1 and NOS2 A), are more profusely expressed in lambs that are resistant to *H. contortus* and *Trichostrongylus colubriformis* infections (31).

Different important domains of RIG-I of *Ovis aries* have been analyzed through in silico studies. The C-terminal regulatory domain is one of the most functional domains in the gene. It binds with the viral RNA, and activation of the RIG-I ATPase by RNA-dependent dimerization occurs. RD type is a zinc-binding domain that is related to GDP/GTP. Similarly, the molecular kinetics of RIG-I contain two N-terminal CARDs and one RNA helicase domain, which help in relaying the signal to the downstream signal adaptor MAVS, which leads to the activation of type I IFN responses (32). MDA5, along with RIG-I, results in the binding of RLRs to the MAVS, which is a signal adaptor that influences the activation of the NFkb gene. Finally, LGP2 plays a role in producing antiviral responses against viruses that are recognized by RIG-I.

Similar studies that correspond to our findings indicate an increase in serum concentrations of IgE, IgA, IgG, TNF-β, IFN-γ, and IL-6 in naturally infected sheep with *Haemonchus* spp. on pastures with two different nutritional conditions (30) by studying the immune response in sheep by significantly higher peripheral eosinophilia.

The binding sites of the Haemonchus protein with the RIG-I protein are analyzed by molecular docking. The binding position of the parasitic protein nd4 has been found in between 515 leucine to 612 tyrosine and 826 isoleucin to 868 leucine. Biologically, nd4 is the core subunit of the mitochondrial respiratory chain NADH dehydrogenase (Complex I) that is believed to belong to the minimal assembly required for catalysis. Complex I functions in the transfer of electrons from NADH to the respiratory chain. The immediate electron acceptor for the enzyme is assumed to be ubiquinone. Domain analysis for RIG-I reveals the polypeptide binding site (RD interface) as nucleotide sites 522, 525-526, 539-540, 543. Other polypeptide binding sites (Helicase domain interface) for RIG-I are 514-515, 518-522. Interestingly, it has been observed that the binding site of RIG-I corresponds with the parasitic proteins (nd4, alpha tubulin, beta tubulin, cystein proteinase, lectin, galectin, pepk) as revealed through in silico studies. The pgp1 protein that binds from Lysine 394 to Phenylalanine 492 possesses ATPase activity. Pgp1 protein, which is also known as a multidrug-resistant protein, removes lipophilic xenobiotic compounds from cells functioning as efflux pumps and also plays a role in the resistance of parasitic nematodes to anthelmintic drugs, such as benzimidazoles and macrocyclic lactones. Several secretory parasitic proteins that play an important role in dominating the host immune system also have been observed at the position between Valine 520 and Asparagine 665. These kinds of proteins either depress the host immune system or instigate the system. It is reported that Haemonchus contortus excretory and secretory proteins (HcESPs) can bind to goat peripheral blood mononuclear cells. HcESPs affected the biological functions of the cell, such as cell proliferation, cytokine production, cell migration, and nitric oxide production of goat PBMCs (33). A gradual decrease in interleukin-4 and interferon-γ was observed in PBMCs; in contrast, there was an increase in the concentration of IL-10 and IL-17 (34). Nitric oxide production was suppressed at different concentrations of HcESPs. Similarly, cell proliferation was also decreased at all concentrations of the parasitic proteins (34). The Glc 5 parasitic protein (Glutamate-gated chloride channel) binds from Glycine 307 to Leucine 868. These chloride channels perform extracellular ligand gated ion channel activity, ion transport, and transmembrane signaling. *H. contortus*, being a highly resistant and polymorphic parasite in livestock, is also resistant to Ivermectin. On the other hand, *Caenorhabditis elegans* enables the study of the effects of polymorphism in resistant parasites. Reports claim that this was tested using glutamate chloride channels that form candidate resistant genes and an ivermectin drug target (35).

α- and β-tubulin, being major constituents of microtubules, bind two moles of GTP, one at an exchangeable site on the alpha chain and one at a nonexchangeable site on the beta chain. They bind from the Histidine 302 position to the Proline 763 position. They play an important role in Haemonchus contortus by providing resistance to Benzimidazole Antihelmintics (36). The parasitic protein Cystein proteinase binds from the Methionine 305 position to Glutamate 748. This protein has a peptidase activity and is responsible for breaking down the host hemoglobin. Haemonchus contortus is of the most highly pathogenic parasite of ruminants, and cystein proteases act as one of the prime targets for vaccine against H. contortus. Being an active protease of the excreatory- secretory product of H. contortus, there is strong probability of its involvement in induction of protective immunity (37). These proteases are also considered as important digestive enzymes in parasitic helminthes.

Phosphoenol pyruvate carboxy kinase is yet another important parasitic protein that binds from the position Arginine 298 to Phenylalanine 882. This is an important enzyme in helminth parasites because it has GTP and metal ion binding activity, and moreover, it catalyzes the reverse reaction of formation of oxaloacetic acid from phosphoenol pyruvate (PEP) rather than in mammals in which the reaction is vice versa. This leads to an important functional difference between the host and the parasite, and it can be predicted as a novel target for antihelminthic drugs. These predictions are subject to experimental validation and discovery of novel chemotherapeutic agents to fight against helminthes, protozoas, etc (38). Last, Galaectin, from position Threonine 330 to Leucine 717 and Lectin from position Histidine 295 and Threonine 462 are the proteins that recognize and bind to specific carbohydrate moieties.

## Conclusion

RIG-I of Ovis aries is characterized for the first time. Some important domains for ovine RIG-I are identified. So far, RIG-I gene has been studied mostly as an agent conferring antiviral immunity. In this current study, we detected the role of RIG-I in conferring immunity against the gastrointestinal nematode Haemonchus contortus. The fact was revealed through the differential mRNA expression profile of RIG-I in Haemonchus-infected with respect to healthy sheep. The fact was later confirmed through in silico studies with molecular docking. Because this is the first report, we identified the binding site for RIG-I with the polypeptide of parasite at 300-750 for most of the protein. Alpha tubulin, Beta tubulin, lectin, galectin, and cysteine protease were predicted to be the most promising binding site with ovine RIG-I. Molecular phylogeny detects sufficient genetic similarity of domestic sheep with humans, next to primates. Hence, sheep may be effectively employed as an animal model for studying parasitic immunology.

## Data Availability Statement

The datasets presented in this study can be found in online repositories. The names of the repository/repositories and accession number(s) can be found here: NCBI [Accession: KX687005].

## Ethics Statement

The animal study was reviewed and approved by Institutional Animal Ethics Committee, West Bengal University of Animal and Fishery Sciences.

## Author Contributions

SB and ArP conducted the research work. ArP and JC planned and designed the research. AbP and ArP conducted bioinformatics analysis. SB and ArP drafted the manuscript. Statistical analysis has been conducted by ArP and PC. ArP, JC, and PC critically revised the manuscript. All authors contributed to the article and approved the submitted version.

## Conflict of Interest

The authors declare that the research was conducted in the absence of any commercial or financial relationships that could be construed as a potential conflict of interest.
